# My Exposure to Violence: translation and cultural adaptation to the BR Portuguese

**DOI:** 10.11606/s1518-8787.2022056004080

**Published:** 2022-08-16

**Authors:** Marcos Clint Leal de Carvalho, Raimunda Hermelinda Maia Macena

**Affiliations:** I Universidade Federal do Ceará Faculdade de Medicina Programa de Pós-Graduação em Saúde Pública Fortaleza CE Brasil Universidade Federal do Ceará. Faculdade de Medicina. Programa de Pós-Graduação em Saúde Pública. Fortaleza, CE, Brasil; II Instituto Federal de Educação, Ciência e Tecnologia do Ceará Fortaleza CE Brasil Instituto Federal de Educação, Ciência e Tecnologia do Ceará. Reitoria. Fortaleza, CE, Brasil

**Keywords:** Adolescent, Exposure to Violence, Surveys and Questionnaires, Translating, Psychometrics

## Abstract

**OBJECTIVE:**

To translate and adapt the My Exposure to Violence instrument for measuring exposure to community violence into Brazilian Portuguese.

**METHODS:**

Psychometric study of translation and cross-cultural adaptation in seven stages: (I) initial translations, (II) synthesis of translations, (III) back-translations, (IV) cross-cultural pre-adaptation, (V) evaluation by committee of judges, (VI) pre-test, and (VII) submission to the original author. In step V eight judges evaluated the instrument’s equivalence of content, and the content validity index was calculated for each item (CVI-I) and for the average of the instrument (CVI-M), considering a CVI-I ≥ 0.78 as excellent and a CVI-M ≥ 0.80 as acceptable and ≥ 0.90 as excellent. The pre-test was conducted with 39 adolescents enrolled in an educational institution in Fortaleza, state of Ceará. The understandability of the items was evaluated.

**RESULTS:**

In step I, two translations were produced with few differences between them. These were resolved in step II, by generating the synthesis version (T12). In step III, the back-translated items reflected the same content as the items in the original version. In step IV, T12 was revised by the authors, who made specific linguistic changes in order to facilitate understanding. In step V, one item (22) presented CVI < 0.78. Due to the relevance of the suggestions, 19 of the 23 items (82.60%) were modified. The CVI-M of the instrument was 0.92. In stage VI, the mean age of the participants was 17.48 years (SD = 1.27). The pre-final version had 21 out of 23 items (91.30%) fully understood by more than 90% of the participants. No changes were made to the final version.

**CONCLUSIONS:**

My Exposure to Violence was cross-culturally adapted into Brazilian Portuguese, and was well understood by the target population. Other psychometric properties, such as reliability and validity, should be evaluated in further studies to strengthen the evidence of the translated and adapted version.

## INTRODUCTION

Violence is recognized as a public health issue^1^. Among the several forms of violence, community violence is considered especially harmful, causing several adverse impacts on the health of individuals who are exposed to it^2^.

Although there is divergence in the literature as to the concept of community violence, it is typically defined and measured by researchers in terms of interpersonal harm or threat of harm within a neighborhood or community, excluding related concepts such as domestic violence, abuse and bullying^3^.

Adolescents are a share of the population particularly vulnerable to exposure to a wide range of types of violence, especially community violence^4^. The prevalence of exposure to community violence (ECV) among adolescents varies in international literature reviews from 3% to 96%. This range may be explained by the use of different measuring instruments, by data collection from different sources, by the approach to different types of exposure to violence, and by actual differences in prevalence due to intrinsic characteristics of communities^5^.

Exposure to community violence has been related to a range of negative outcomes among adolescents, such as mental health problems^6^, depression^7^, anxiety^8^, post-traumatic stress disorder^9^, aggression^10^ and delinquency^11^. Studies using neuroimaging have shown an association between ECV and reduced gray matter volume and lower IQ^12^, and ECV in early adolescence as a predictor of lower hippocampal and amygdala volume in late adolescence, which may imply impairments in learning, memory, and emotion processing^13^.

The study on exposure to community violence among adolescents in Brazil is still quite fragmented and virtually restricted to capital cities in the South and Southeast of Brazil^4^. Moreover, no instrument was found in Portuguese language to measure this exposure that had undergone a rigorous process of translation and cross-cultural adaptation, i.e., based on a method well-established in literature, and describing in detail the steps followed. For example, Zavaschi et al.^14^ evaluated the prevalence of ECV of adolescents in Porto Alegre, Rio Grande do Sul, using the Survey of Children’s Exposure to Community Violence (SCECV)^15^. The only information available regarding the process of translation and cross-cultural adaptation was that the English instrument had been translated into Portuguese and back-translated by two independent translators. In Rio de Janeiro, a study^5^ evaluated the association between ECV and post-traumatic stress disorder in children and adolescents aged nine to 16 years, using a translation of the Things I have seen and heard^16^ instrument. This instrument is an adaptation of the aforementioned SCECV, designed for children aged six to eight years and that mainly assesses indirect exposure to violence. Furthermore, both instruments include items of questionable characterization as community violence, such as seeing drug dealing, seeing a dead body (without specification of cause of death by violence) or suffering accidents. Two other studies^17,18^ used items taken from an instrument developed in Brazil, called the Brazilian Youth Questionnaire^19^, to measure exposure to community violence, consisting of only five items assessing the occurrence of threat or humiliation, punching or beating, assault with objects, forced intimate touching, and rape, which is a very narrow characterization of community violence.

Thus, this study proposed to translate and adapt into Brazilian Portuguese the My Exposure to Violence (My ETV) instrument to measure exposure to community violence^20,21^. My ETV was originally developed for use in Chicago, USA^20^, and was later adapted to better measure specifically exposure to community violence^21^. It consists of dichotomous items (yes/no answers) and addresses CVE in the fields of (1) direct victimization, (2) witnessing, and (3) becoming aware of an episode of violence. It was, thus, divided into three subscales, the first with seven items and the others with eight items each. It is considered one of the most robust psychometrically validated instruments for measuring exposure to violence, one of the few to assess the three categories of exposure^3^ and has been widely used in the USA^11,22^ and in other countries^23,24^.

In a validation study, the instrument was applied to 1,871 children and adolescents aged nine to 19 years, and a Cronbach’s alpha of 0.82 was found. A confirmatory factor analysis indicated the adequacy of the hypothesis of subdivision of exposure to community violence into three exposure pathways. Finally, the latent score of ECV, calculated by applying an item response theory model, showed strong correlations with variables shown in the literature to be associated with ECV, such as measures of anxiety/depression, delinquency, aggressiveness, and sociodemographic characteristics^21^.

## METHODS

This is a psychometric study that consisted of the translation and cross-cultural adaptation of the self-applied version of the instrument for measuring exposure to community violence, My ETV. The use of My ETV in this work was authorized by one of its main authors.

The translation and cross-cultural adaptation process was based on the model proposed by Beaton et al.^25^, consisting of the following steps: (I) initial translation, (II) synthesis of translations, (III) back translation, (IV) cross-cultural pre-adaptation, (V) review by committee of judges, (VII) pre-test, and (VIII) submission to the authors of the original version, and step IV was included by the authors of the current article.

Two translations of My ETV (T1 and T2) into Portuguese were independently performed by two bilingual Portuguese native-speaking translators (stage I). One translator was informed about the objectives and concepts addressed by the instrument, while the other was a professional English-Portuguese literary translator with no experience in health research (“naïve” translator). After T1 and T2, the translators held a videoconference, mediated by an external observer with experience in translation and cross-cultural adaptation, to discuss the differences between translations and get a synthesis version (T12) (step II). Next, T12 was submitted to two professional translators with English mother tongue (one South African and one American, living in Brazil for seven and 26 years, respectively), with no background in the field of health, and who did not know the original instrument, for the back translation process. The BT1 and BT2 versions (stage III) were then produced.

Before the review by the committee of judges, adding a stage to the method proposed by Beaton et al^25^, the authors revised the T12 summary version (stage IV), making some changes, such as in word order, seeking to bring the subject closer to the verb and changes in vocabulary and idiomatic expressions. This procedure aimed to facilitate the understanding of the questionnaire by its target audience, and optimize the review process by the judges. The new revision was named cross-cultural pre-adaptation (pre-CCA).

Once the translations phase was finished, the review by the judges committee took place (step V). Invitations were e-mailed to 45 potential judges, who met at least one of the following criteria: (1) have an English Language and/or Linguistics degree; (2) be a researcher with experience in development and/or cross-cultural adaptation of research instruments (methodologist), with self-reported mastery of the English language; and (3) be a researcher with experience in the area of violence, with self-reported mastery of the English language.

Each judge who agreed to participate in the study received an e-mail with the informed consent form (ICF), the instructions for the evaluation, the questionnaire in its original, T1, T2, T12, BT1, BT2, and pre-CCA versions, and the evaluation instrument for semantic, idiomatic, cultural, and conceptual equivalence of the pre-CCA version of the My ETV. The judges’ evaluation instrument, in Microsoft Office 365^®^ software text format, consisted of the pre-CCA version alongside the original, with each item having a specific scoring field for the four types of equivalence, scored on a Likert-type scale with the following options: 1 = no equivalence, 2 = little equivalence, 3 = medium equivalence, 4 = a lot of equivalence, and 5 = total equivalence. Space was provided for suggestions, used in items scoring less than four. The judges were instructed to consider semantic equivalence as the maintenance of the meaning of the words between the original instrument and the translated one, idiomatic equivalence as the equivalence of colloquialisms or idiomatic expressions, cultural equivalence as the coherence of the translation with the culture of the target population and conceptual equivalence as the evaluation of whether the translated terms represent the same concept as the original terms^26^.

The agreement between the judges regarding the adequacy of the equivalences of each item was evaluated by means of the content validity index (CVI), one of the most used methods^27^, with the formula: (number of judges who scored 4 or 5) / (total number of judges), from which the CVI of each item for each of the four equivalences and the overall CVI of each item (CVI-I), as the arithmetic mean of the CVI of the four equivalences, were calculated. Finally, the mean CVI of the instrument (CVI-M) was calculated, as the arithmetic mean of the CVI-I of each of the items. A CVI-I ≥ 0.78 was considered as excellent and a CVI-M ≥ 0.80 as acceptable and ≥ 0.90 as excellent^28^. The judges’ suggestions were then compiled and evaluated by the authors, who reached consensus on which changes to make, thus arriving at the pre-final version, used in the next step.

The pre-test (step VI) was performed with adolescents enrolled in a federal institution that offers integrated technical and higher education courses in Fortaleza, Ceará. This population was chosen due to the heterogeneous sociocultural profile of the students, allowing the evaluation of the instrument by different strata of the target population. Participants were randomly selected among all the students aged up to 19 years old at the time of the study (adolescents). The selected participants received a link to a questionnaire by e-mail, containing the ICF (older than 18 years) or the Consent Form and the ICF for the tutor (younger than 18 years), and the data collection instrument itself, consisting of the pre-final version of My ETV in Portuguese, evaluation of the understanding of each item, an evaluation of the instrument as a whole, and questions for sociodemographic characterization. To evaluate the understanding of the items individually, each item was exposed separately and participants were offered three response options: “I did not understand the question”, “I more or less understood the question”, and “I understood the question”. Participants were asked to indicate how they would rewrite the items marked with the first or second options or make suggestions, and a specific field was provided for this. The link was sent to 800 students, with a target of 30 to 40 participants from February 8, 2021 to April 8, 2021.

The target audience’s suggestions were then compiled and evaluated by the authors. The final version was then submitted, with all the documentation produced throughout all the steps, to the author of the original instrument (step VII).

The study followed the ethical principles of research involving human beings of Resolution 466/2012 of the National Health Council, and was approved by the research ethics committees of the *Universidade Federal do Ceará* and the *Instituto Federal de Educação, Ciência e Tecnologia* of Ceará under opinions n° 4.026.654 (CEP/UFC) and 4.328.491 (CEP/IFCE).

## RESULTS

For the preparation of the synthesis version (T12), the structuring of the items in almost all cases was done by combining both translations provided. The discussion among the translators began with the items standardization, i.e., to ensure that expressions used more than once were always translated in the same way. In this sense, 21 of the 23 items contain the expression “in your whole life” and all contain the term “ever”. Translator 1 chose to literally follow the original, using for item 1, for example, “*Em toda sua vida, você alguma vez*…”. Translator 2, on the other hand, compressed the expression, using “*Você alguma vez na vida*”. For the synthesis, it was considered that the compact version maintained the meaning of the original, with the advantage of making the reading less boring.

Only one relevant semantic divergence was found between T1 and T2. In item 1, the part “could really get hurt” was translated as “*poderia se machucar seriamente*” by translator, 1 and as “*realmente corria risco de se machucar*” by translator 2. For the synthesis version, the translators concluded that the solution present in T2 proved to be more appropriate to the intent of the original item, i.e., the term “really” indicating high probability of suffering some damage, and not the severity of the damage.

The back-translations showed some differences in relation to the original. For example, no back-translated item started with “In your whole life”, which was already expected from the decisions made in the construction of T12. Even so, the back-translated items reflected the same content as the items in the original version, and the back-translations fulfilled the purpose of validity checking and detecting possible conceptual errors and gross inconsistencies in T12^25^.

In the pre-CCA stage, the authors made changes to 18 of the 23 items (78.26%), such as replacing the expression “*Você alguma vez na vida viu*” by “*Alguma vez na vida você viu*” and the term “*bastão*” by “*pedaço de pau*”.

**Table 2:** Understanding of the items of *Minha Exposição à Violência*. Fortaleza, Ceará, Brazil, 2021.

Item	I did not understand the question n (%)	I more or less understood the question n (%)	I understood the question n (%)
1	0	0	39 (100)
2	0	1 (2.56)	38 (97.44)
3	0	2 (5.13)	37 (94.87)
4	0	0	39 (100)
5	0	0	39 (100)
6	0	1 (2.56)	38 (97.44)
7	0	0	39 (100)
8	0	0	39 (100)
9	2 (5.13)	2 (5.13)	35 (89.74)
10	2 (5.13)	2 (5.13)	35 (89.74)
11	0	0	39 (100)
12	0	0	39 (100)
13	0	1 (2.56)	38 (97.44)
14	0	1 (2.56)	38 (97.44)
15	0	0	39 (100)
16	1 (2.56)	0	38 (97.44)
17	0	0	39 (100)
18	0	0	39 (100)
19	0	2 (5.13)	37 (94.87)
20	0	0	39 (100)
21	0	1 (2.56)	38 (97.44)
22	0	0	39 (100)
23	0	1 (2.56)	38 (97.44)

From the total of 45 professionals invited to compose the committee, 15 agreed to participate, but only 8 returned the evaluation instrument by the stipulated deadline. The participating judges were professionals from the areas of Nursing, Physiotherapy, Psychology and English Language, all PhD and professors, of which three met inclusion criterion “a”, one met criterion “b”, two met criterion “c”, and two met both criteria “b” and “c”. In the evaluation by the committee of judges, the pre-CCA version showed excellent semantic, idiomatic, conceptual, and acceptable cultural equivalences. Only one item (22) showed CVI-I < 0.78. The CVI-M of the instrument was 0.92, considered excellent (Table 1).

**Table 1:** Content validity index by equivalences, by items and total. Fortaleza, Ceará, Brazil, 2021.

	Semantic	Idiomatic	Cultural	Conceptual	CVI-I
Item 1	1	1	1	1	1
Item 2	1	1	1	1	1
Item 3	1	1	0.88	1	0.97
Item 4	1	1	0.88	1	0.97
Item 5	1	1	1	0.88	0.97
Item 6	1	1	0.88	1	0.97
Item 7	0.88	1	0.88	0.88	0.91
Item 8	0.88	1	0.75	0.88	0.88
Item 9	0.88	0.88	0.75	1	0.88
Item 10	1	1	0.75	1	0.94
Item 11	0.88	0.88	0.88	0.88	0.88
Item 12	1	1	0.75	0.88	0.91
Item 13	0.88	1	0.88	1	0.94
Item 14	0.88	1	0.88	1	0.94
Item 15	1	1	1	1	1
Item 16	0.88	1	1	1	0.97
Item 17	1	1	1	1	1
Item 18	1	1	1	1	1
Item 19	0.88	0.88	0.75	0.88	0.84
Item 20	0.88	0.88	0.75	0.88	0.84
Item 21	0.75	0.88	0.75	0.75	0.78
Item 22	0.75	0.88	0.63	0.75	0.75
Item 23	0.88	0.88	0.75	0.75	0.81
**Total instrument**	**0.93**	**0.96**	**0.86**	**0.93**	**0.92**

CVI-I: content validity index for each item.

The judges’ suggestions were mostly aimed at achieving greater cultural equivalence. Of the eight judges, only two did not suggest any changes to the instrument. Item 22, for having obtained IVC-I < 0.78, would be the only one to necessarily have to be modified. However, due to the high pertinence of the suggestions, 19 of the 23 items (82.60%) underwent some modification in the pre-final version, used in the pre-test. Box 1 presents the original, pre-CCA and pre-final versions of My ETV.

**Box 1:** Original versions, pre-CCA and final of My Exposure to Violence. Fortaleza, Ceará, Brazil, 2021.

Original	Pre-CCA	Final version
1. In your whole life, have you EVER seen someone else get chased when you thought they could really get hurt?	*Alguma vez na vida você viu alguma outra pessoa ser perseguida e achou que ela realmente corria risco de se machucar? (=)*	*Alguma vez na vida você viu alguma outra pessoa ser perseguida e achou que ela realmente corria risco de se machucar? (=)*
2. In your whole life, have you EVER been chased when you thought that you could really get hurt?	*Alguma vez na vida você foi perseguido e achou que realmente corria risco de se machucar?*	*Alguma vez na vida você foi* **perseguido(a)** *e achou que realmente corria risco de se machucar?*
3. In your whole life, have you EVER seen someone else get hit, slapped, punched, or beaten up? This does not include when they were playing or fooling around.	*Alguma vez na vida você viu alguma outra pessoa apanhar, levar tapa, soco ou surra? Isso não inclui situações de brincadeira ou gozação.*	*Alguma vez na vida você viu alguma outra pessoa apanhar, levar* **um tapa, um soco ou uma surra***?**Não considerar situações em que os envolvidos estão rindo ou brincando.*
4. In your whole life, have you EVER been hit, slapped, punched, or beaten up? Again, this does not include when you were playing or fooling around.	*Alguma vez na vida você apanhou, levou tapa, soco ou surra? Mais uma vez, isso não inclui situações de brincadeira ou gozação.*	*Alguma vez na vida você apanhou, levou* **um tapa, um soco ou uma surra***? Mais uma vez,**não considerar situações em que os envolvidos estão rindo ou brincando.*
5. In your whole life, have you EVER seen someone else get attacked with a weapon, like a knife or bat? This does not include getting shot or shot at.	*Alguma vez na vida você viu alguma outra pessoa ser atacada com uma arma, como uma faca ou pedaço de pau? Isso não inclui ser baleado ou ter tiros disparados contra si.*	*Alguma vez na vida você viu alguma outra pessoa ser atacada com uma arma, como uma faca ou pedaço de pau?* **Isso não inclui a pessoa ser baleada ou ter tiros disparados contra ela.**
6. In your whole life, have you EVER been attacked with a weapon, like a knife or bat? Again, this does not include getting shot or shot at.	*Alguma vez na vida você foi atacado com uma arma, como uma faca ou um pedaço de pau? Mais uma vez, isso não inclui ser baleado ou ter tiros disparados contra si.*	*Alguma vez na vida você foi* **atacado(a)** *com uma arma, como uma faca ou um pedaço de pau? Mais uma vez, isso não inclui ser baleado ou ter tiros disparados contra si.*
7. In your whole life, have you EVER seen someone else get shot? This doesn’t include seeing someone shot with a BB gun or any type of toy gun.	*Alguma vez na vida você viu alguma outra pessoa levar um tiro? Isso não inclui ver alguém levar um tiro de arma de chumbinho ou de qualquer tipo de arma de brinquedo. (=)*	*Alguma vez na vida você viu alguma outra pessoa levar um tiro? Isso não inclui ver alguém levar um tiro de arma de chumbinho ou de qualquer tipo de arma de brinquedo. (=)*
8. In your whole life, have you EVER been shot? Again, this doesn’t include being shot with a BB gun or any type of toy gun.	*Alguma vez na vida você levou um tiro? Mais uma vez, isso não inclui levar um tiro de arma de chumbinho ou de qualquer tipo de arma de brinquedo. (=)*	*Alguma vez na vida você levou um tiro? Mais uma vez, isso não inclui levar um tiro de arma de chumbinho ou de qualquer tipo de arma de brinquedo. (=)*
9. In your whole life, have you EVER seen someone else get shot AT, but not actually wounded?	*Alguma vez na vida você viu atirarem em alguma outra pessoa, mas sem essa pessoa ser mesmo ferida?*	*Alguma vez na vida você viu atirarem em alguma outra pessoa, mas sem essa pessoa* **ter sido ferida?**
10. In your whole life, have you EVER been shot AT, but not actually wounded?	*Alguma vez na vida atiraram em você, mas sem você ter sido mesmo ferido?*	*Alguma vez na vida atiraram em você, mas* **sem você ter sido ferido(a)?**
11. In your whole life, have you EVER seen someone else get killed as a result of violence, like being shot, stabbed, or beaten to death?	*Alguma vez na vida você viu alguma pessoa ser morta por meio de violência, como levar um tiro, ser esfaqueada ou espancada até a morte?*	*Alguma vez na vida você viu alguma pessoa ser morta* **como consequência** *de violência, como levar um tiro, ser esfaqueada ou espancada até a morte?*
12. In your whole life, have you EVER been sexually assaulted, molested, or raped?	*Alguma vez na vida você sofreu abuso sexual, foi molestado ou estuprado?*	*Alguma vez na vida você sofreu abuso sexual, foi* **molestado(a) ou estuprado(a***)**ou lhe tocaram nas partes intimas sem que você quisesse?*
13. OTHER THAN WHAT YOU HAVE ALREADY TOLD ME, have your EVER seen someone threaten to seriously hurt another person? This includes being threatened with a weapon.	*ALÉM DO QUE VOCÊ JÁ me CONTOU, alguma vez na vida você viu alguém ameaçar machucar seriamente outra pessoa? Isso inclui ser ameaçado com uma arma.*	*ALÉM DO QUE VOCÊ JÁ me CONTOU, alguma vez na vida você viu alguém ameaçar ou machucar seriamente outra pessoa? Isso inclui**ameaça com qualquer tipo de arma.*
14. OTHER THAN WHAT YOU HAVE ALREADY TOLD ME, has someone EVER threatened to seriously hurt you? Again, this includes being threatened with a weapon.	*ALÉM DO QUE VOCÊ JÁ me CONTOU, alguma vez na vida alguém ameaçou machucar você seriamente? Mais uma vez, isso inclui ser ameaçado com uma arma.*	*ALÉM DO QUE VOCÊ JÁ me CONTOU, alguma vez na vida alguém ameaçou machucar você seriamente? Mais uma vez, isso inclui**ameaça com qualquer tipo de arma.*
15. In your whole life, have you EVER been told that someone you knew had been shot, but not killed?	*Alguma vez na vida lhe contaram que alguém que você conhecia tinha levado um tiro, mas não foi morto?*	*Alguma vez na vida* **te** *contaram que alguém que você conhecia tinha levado um tiro, mas não* **tinha morrido***?*
16. In your whole life, have you EVER been told that someone you knew had been killed?	*Alguma vez na vida lhe contaram que alguém que você conhecia tinha sido assassinado?*	*Alguma vez na vida* **te** *contaram que alguém que você conhecia tinha sido* **assassinado(a)***?*
17. In your whole life, have you EVER been told that someone you knew had been raped?	*Alguma vez na vida lhe contaram que alguém que você conhecia tinha sido estuprado(a)? (=)*	*Alguma vez na vida te contaram que alguém que você conhecia tinha sido estuprado(a)? (=)*
18. In your whole life, have you EVER seen someone else sexually assaulted, molested, or raped?	*Alguma vez na vida você viu alguma outra pessoa sofrer abuso sexual, ser molestado ou estuprado?*	*Alguma vez na vida você viu alguma outra pessoa sofrer abuso sexual, ser* **molestado(a)** *ou* **estuprado(a)***ou tocarem nas partes intimas dela sem que ela quisesse?*
19. In your whole life, have you EVER been told that someone you knew had been chased when you thought they could really get hurt	*Alguma vez na vida lhe contaram que alguém que você conhecia tinha sido perseguido e achou que essa pessoa realmente correu risco de se machucar?*	*Alguma vez na vida* **te** *contaram que alguém que você conhecia tinha sido* **perseguido(a) e você achou que essa pessoa realmente poderia se machucar***?*
20. In your whole life, have you EVER been told that someone you knew had been slapped, punched, or beaten up? This does not include when they were playing or fooling around.	*Alguma vez na vida lhe contaram que alguém que você conhecia tinha levado tapa, soco ou surra? Isso não inclui situações de brincadeira ou gozação.*	*Alguma vez na vida* **te** *contaram que alguém que você conhecia tinha levado* **um tapa, um soco ou uma surra***?**Não considerar situações em que os envolvidos estão rindo ou brincando.*
21. In your whole life, have you EVER been told that someone you knew had been attacked with a weapon, like a knife or bat? This does not include getting shot or shot at.	*Alguma vez na vida lhe contaram que alguém que você conhecia tinha sido atacado com uma arma, como uma faca ou pedaço de pau? Isso não inclui ser baleado ou ter tiros disparados contra si.*	*Alguma vez na vida* **te** *contaram que alguém que você conhecia tinha sido* **atacado(a)** *com uma arma, como uma faca ou pedaço de pau? Isso não inclui ser* **baleado(a)** *ou* **ter tiros disparados contra ela**.
22. In your whole life, have you EVER been told that someone you knew had been shot AT, but not actually wounded?	*Alguma vez na vida lhe contaram que atiraram em alguém que você conhecia, mas sem essa pessoa ser mesmo ferida?*	*Alguma vez na vida* **te** *contaram que atiraram em alguém que você conhecia,* **mas sem essa pessoa ter sido ferida***?*
23. OTHER THAN WHAT YOU HAVE ALREADY TOLD ME, in your whole life, have you EVER been told that someone had threatened to seriously hurt someone you knew? This includes being threatened with a weapon.	*ALÉM DO QUE VOCÊ JÁ me CONTOU, alguma vez na vida lhe contaram que alguém havia ameaçado machucar seriamente alguém que você conhecia? Isso inclui ameaças com arma.*	*ALÉM DO QUE VOCÊ JÁ me CONTOU, alguma vez na vida* **te** *contaram que* **alguma pessoa** *havia ameaçado machucar seriamente alguém que você conhecia? Isso inclui**ameaça com qualquer tipo de arma.*

Pre-CCA: cross-cultural pre-adaptation.

Note: in the final version, bold indicates a suggestion accepted literally, while underlined indicates a change made by the authors to incorporate the suggestion. The sign (=) indicates that no changes were made.

The formatting in capital letters was kept only for the expression “*ALÉM DO QUE VOCÊ JÁ me CONTOU*” (items 13, 14, and 23) by decision of the authors.

The mean age of the 39 pre-test participants was 17.48 years (SD = 1.27), with minimum of 15 and maximum of 19 years. Most were female (51.28%), brown (71.79%), Catholic (41.03%), single (92.31%), and lived in Fortaleza (74.36%).

Of the 23 items in the pre-final version, 21 (91.30%) were fully understood by more than 90% of the participants. The items with the most difficulty in understanding were items 9 and 10, but both were reported as fully understood by 35 students (89.74%), as shown in Box 2.

**Box 2:** Suggestions made by the participants of the pre-test. Fortaleza, Ceará, 2021.

Item	Suggestions
2. Have you ever been harassed in your life and felt that you were actually in danger of getting hurt?	Have you ever been exposed in your life to any kind of violence, either physical or psychological? Or have you ever felt physically or psychologically attacked?
5. Have you ever seen another person attacked with a weapon, such as a knife or a piece of wood? This does not include the person being shot or having shots fired at them.	I understood; however I would put the observation in parentheses.
9. Have you ever in your life seen someone shooting another person, but without that person being injured?	Have you ever seen someone try to shoot a person, but because they didn’t hit him or her, the person wasn’t hurt? Have you ever witnessed an attempted murder by firearm?
10. Have you ever been shot at in your life, but without being wounded?	Have you ever been shot at, but you weren’t hurt because you weren’t hit? Have you ever had a traumatic experience where someone tried to hurt you with a firearm?
13. IN ADDITION TO WHAT YOU HAVE TOLD ME, have you ever seen someone threaten or seriously injure another person? It includes threatening with any kind of weapon.	I understand what you mean, but I haven’t told you anything, so it couldn’t be “IN ADDITION TO WHAT YOU HAVE TOLD ME”.
14. IN ADDITION TO WHAT YOU HAVE TOLD ME, has anyone in your life ever seriously threatened to hurt you? Again, this includes threatening with any kind of weapon.	I understand what you mean, but I haven’t told you anything, so it couldn’t be “IN ADDITION TO WHAT YOU HAVE TOLD ME”.
19. Have you ever been told in your life that someone you knew was being chased, and you thought that this person might really get hurt?	Have you ever been told in your life that someone you knew had been persecuted, and you thought that this person might have been hurt?
23. IN ADDITION TO WHAT YOU HAVE TOLD ME, have you ever in your life been told that someone had threatened to seriously harm someone you knew? It includes threatening with any kind of weapon.	I understand what you mean, but I haven’t told you anything, so it couldn’t be “IN ADDITION TO WHAT YOU HAVE TOLD ME”.

Note: The formatting in capital letters was kept only for the expression “IN ADDITION TO WHAT YOU HAVE TOLD ME” [“*ALÉM DO QUE VOCÊ JÁ me CONTOU*”] (items 13, 14, and 23) by decision of the authors.

Only a small portion of the participants 6 (15.38%) made suggestions for changes in the items. Of the 23 items, eight (34.78%) received suggestions/criticisms. Box 2 presents all the comments from the participants.

The authors evaluated that the suggestions were either impertinent (items 13, 14 and 23) or would imply in substantial changes in the content of the items (item 2 and item 10b) or presented an excessively technical formulation (item 9b). Regarding the others, although pertinent, it was considered that they differed very little from the items presented. Thus, no changes were made to the items in the pre-final version.

In the general evaluation, the great majority of the participants, 31 (79.49%), considered that they could answer the instrument easily, only 12 (30.77%) considered the questionnaire long, and only 11 (28.21%) considered it repetitive. Most of the sample, 32 (82.05%) and 37 (94.87%), thought that the answers of the instrument let one know if an adolescent has already suffered some kind of violence, and if an adolescent knows someone who has already suffered some kind of violence, respectively.

Considering the positive evaluation of the instrument as a whole, and the high understanding of its items by the participants of the pre-test, the process of translation and adaptation to the Brazilian cultural context of My ETV, with the title *Minha Exposição à Violência*, was considered completed, and the pre-final version was kept as the final version. All versions produced throughout the process were submitted to one of the original authors, who approved the final version in Portuguese and congratulated us for maintaining the conceptual intent of the items.

## DISCUSSION

The translation and cross-cultural adaptation of the instrument into Brazilian Portuguese measuring exposure to community violence, My ETV, was carried out, obtaining excellent semantic, idiomatic, and conceptual equivalence, and acceptable cultural equivalence by judges, besides the high understanding of the items by the target population.

The methodological process of preparing a research instrument for application in a population with a different language, culture, concepts, and experiences from the one for which it was originally developed requires that a rigorous and standardized script be followed to obtain a reliable translated instrument^29^. In this study, we followed the steps proposed by Beaton et al.^25^ a reference used in Brazil and internationally^31^ in most cross-cultural adaptation studies, added with a step called pre-CCA, which consisted of a preliminary analysis of linguistic and cultural aspects.

In step I, translations were done independently by two mother-tongue translators of the target language (Brazilian Portuguese), and synthesized in the T12 version (step II). Selecting qualified translators is crucial to obtain high-quality translations. Matching informed translator and naive translator is necessary to ensure that the translated versions cover both technical aspects and reflect the spoken language and its cultural nuances^29^.

Back-translation serves as an additional quality control check, aimed at verifying that the synthesis version reflects the content of the original instrument, not assuming that they remain literally identical, but that they keep conceptual equivalence^32^. In stage III, back-translations did not present major discrepancies between them or in relation to the original.

A stage was added to the script proposed by Beaton et al.^25^, entitled pre-CCA, consisting of the review of the T12 summary version (stage IV) before review by the committee of judges. Additions and modifications to cross-cultural adaptation processes are common, and generally well-accepted as long as they are acknowledged and described in detail^33^.

In stage V, the committee of judges reached the minimum composition suggested by Beaton et al.^25^, including linguists, methodologists and specialists in the area of the construct, consisting of professionals with high expertise. This enabled generating highly pertinent suggestions, accepted whenever possible, aiming to obtain the greatest possible relevance and understandability of the items, thus ensuring an instrument well-adapted to the Brazilian context.

This process was positively reflected in the pre-test stage. Few suggestions were made and, considering the high rate of understanding of the items, no new changes were made to the instrument. The application to a sample with different profiles of adolescents ensured the results of the evaluation on the understanding of the instrument. Moreover, most participants considered the instrument neither long nor repetitive, which is relevant because these are factors that may be associated with a lower response rate^34^. Thus, My ETV was translated and cross-culturally adapted into Brazilian Portuguese. This is the first step for further validation of the instrument and application on larger samples of adolescents.

The contributions of this study are relevant given the scarcity of instruments properly adapted into Portuguese in the field of community violence. Some studies have assessed ECV using other instruments. Generally there are no descriptions of the translation and cross-cultural adaptation processes and/or the instruments used do not cover the concept of community violence in its three routes of exposure (direct victimization, witnessing and becoming aware of an episode of violence).

No research was found that applied any instrument to measure exposure to community violence among adolescents in the Northeast of Brazil. It is also noteworthy that My ETV is one of the few instruments to assess the three categories of ECV, and that, until the completion of this study, no cross-cultural adaptation study of My ETV had been made in Brazil or any Portuguese version of the instrument, highlighting its contribution toward making the Brazilian version of My ETV available for use and validation in several research contexts.

As limitations of this study, we could mention the virtual data collection. Both in the evaluation by judges and in the pre-test, face-to-face meetings could have generated a greater wealth of information. This possibility was made unfeasible by the context of the SARS-CoV-2 pandemic. However, we believe that the benefits of virtual collection, such as low cost, time optimization, and the opportunity to answer the questionnaire at a chosen moment, with more time for reflection, outweighed the possible limitations.

## CONCLUSIONS

Community violence is a public health problem that mainly impacts adolescents, but there was no adequate instrument in Portuguese language to measure exposure to community violence. The present study performed the translation and cross-cultural adaptation into Brazilian Portuguese of the My ETV measuring instrument, following the most recommended protocol used in the world literature. Other measurement properties, such as reliability and criterion and construct validity, should be evaluated in further studies to strengthen the evidence of the translation and adaptation into Brazilian Portuguese.
