# Supplementary material for: My Exposure to Violence: translation and cultural adaptation to the BR Portuguese
Source: Rev Saude Publica. 2022 Aug 16;56:77. doi: 10.11606/s1518-8787.2022056004080 (PMC9388061; doi:10.11606/s1518-8787.2022056004080)
Supplement: Translation

# Translation: My Exposure to Violence: tradução e adaptação transcultural para o português brasileiro

**Authors:** Marcos Clint Leal de Carvalho, Raimunda Hermelinda Maia Macena

**Affiliations:** I Brasil Universidade Federal do Ceará. Faculdade de Medicina. Programa de Pós-Graduação em Saúde Pública. Fortaleza, CE, Brasil; II Brasil Instituto Federal de Educação, Ciência e Tecnologia do Ceará. Reitoria. Fortaleza, CE, Brasil

**Keywords:** Adolescente, Exposição à Violência, Inquéritos e Questionários, Tradução, Psicometria

## Abstract

**OBJETIVO:**

Traduzir e adaptar para português brasileiro o instrumento de aferição de exposição à violência comunitária, *My Exposure to Violence.*

**MÉTODOS:**

Estudo psicométrico de tradução e adaptação transcultural em sete etapas: (I) traduções iniciais, (II) síntese das traduções, (III) retrotraduções, (IV) pré-adaptação transcultural, (V) avaliação por comitê de juízes, (VI) pré-teste e (VII) submissão ao autor original. Na etapa V oito juízes avaliaram a equivalência de conteúdo do instrumento, sendo calculado o índice de validade de conteúdo para cada item (IVC-I) e para a média do instrumento (IVC-M), considerando-se um IVC-I ≥ 0,78 como excelente e um IVC-M ≥ 0,80 como aceitável e ≥ 0,90 como excelente. O pré-teste foi realizado com 39 adolescentes matriculados em uma instituição de ensino em Fortaleza, no Ceará, sendo avaliada a compreensibilidade dos itens.

**RESULTADOS:**

Na etapa I, duas traduções foram produzidas, com poucas diferenças entre si. Estas foram dirimidas na etapa II, gerando a versão síntese (T12). Na etapa III, os itens retrotraduzidos refletiram o mesmo conteúdo dos itens da versão original. Na etapa IV, ocorreu a revisão de T12 pelos autores, que realizaram modificações linguísticas específicas, de modo a facilitar o entendimento. Na etapa V, um item (22) apresentou IVC < 0,78. Devido a pertinência das sugestões, 19 dos 23 itens (82,60%) sofreram modificações. O IVC-M do instrumento foi 0,92. Na etapa VI, a média de idade dos participantes foi de 17,48 anos (DP = 1,27). A versão pré-final teve 21 dos 23 itens (91,30%) totalmente compreendidos por mais de 90% dos participantes. Não foram realizadas alterações na versão final.

**CONCLUSÕES:**

O *My Exposure to Violence* foi adaptado transculturalmente para o português brasileiro, tendo sido bem compreendido pela população alvo. Outras propriedades psicométricas, como confiabilidade e validade, devem ser avaliadas em estudos posteriores para fortalecer as evidências da versão traduzida e adaptada.

## INTRODUÇÃO

A violência é reconhecidamente um problema de saúde pública^1^. Entre as várias formas de violência, a violência comunitária é considerada especialmente danosa, causando diversos impactos adversos na saúde dos indivíduos que são expostos a ela^2^.

Embora haja divergências na literatura quanto ao conceito de violência comunitária, ela é geralmente definida e aferida pelos pesquisadores em termos de danos ou ameaça de danos interpessoais dentro de uma vizinhança ou comunidade, excluindo-se conceitos relacionados, como violência doméstica, maus tratos e bullying^3^.

Os adolescentes são uma parcela da população que é particularmente vulnerável à exposição aos mais variados tipos de violência, especialmente à violência comunitária^4^. A prevalência da exposição à violência comunitária (EVC) em adolescentes varia em revisões de literatura internacionais de 3% a 96%, podendo tal amplitude de variação ser explicada pela utilização de diferentes instrumentos de aferição, pela coleta de dados a partir de diferentes fontes, pela abordagem de diferentes tipos de exposição à violência e por reais diferenças de prevalência em virtude de características intrínsecas das comunidades^5^.

A exposição à violência comunitária tem sido relacionada a uma gama de desfechos negativos entre os adolescentes, como problemas de saúde mental^6^, depressão^7^, ansiedade^8^, transtorno de estresse pós-traumático^9^, agressão^10^ e delinquência^11^. Estudos utilizando neuroimagem mostraram associação entre EVC e redução de volume da substância cinzenta e menor QI^12^ e EVC no início da adolescência como fator preditor de menor volume do hipocampo e da amígdala ao final da adolescência, o que pode implicar em prejuízos na aprendizagem, memória e no processamento de emoções^13^.

O estudo sobre a exposição à violência comunitária entre adolescentes no Brasil é ainda bastante fragmentado e praticamente restrito a capitais do Sul e Sudeste do país^4^. Além disso, não foi localizado em língua portuguesa nenhum instrumento de aferição dessa exposição que tivesse sido submetido a processo de tradução e adaptação transcultural rigoroso, ou seja, que se baseasse em um método bem estabelecido na literatura e descrevesse pormenorizadamente as etapas seguidas. Por exemplo, Zavaschi et al.^14^ avaliaram a prevalência de EVC de adolescentes em Porto Alegre, no Rio Grande do Sul, utilizando o *Survey of Children’s Exposure to Community Violence* (SCECV)^15^. As únicas informações disponibilizadas quanto ao processo de tradução e adaptação transcultural foram de que o instrumento em inglês fora traduzido para o português e submetido a retrotradução por dois tradutores independentes. No Rio de Janeiro, pesquisa^5^ avaliou a associação entre EVC e transtorno de estresse pós-traumático em crianças e adolescentes com idades entre nove a 16 anos, utilizando uma tradução do instrumento *Things I have seen and heard* ^16^, o qual se trata de uma adaptação do já citado SCECV, voltado para a aplicação em crianças de seis a oito anos de idade e que avalia principalmente exposição indireta à violência, não sendo disponibilizadas informações quanto ao processo de tradução e adaptação transcultural. Ademais, ambos os instrumentos incluem itens de caracterização questionável como violência comunitária, como ver tráfico de drogas, ver um cadáver (sem especificação de causa da morte por violência) ou sofrer acidentes. Outros dois trabalhos^17,18^ utilizaram itens retirados de um instrumento desenvolvido no Brasil, chamado Questionário da Juventude Brasileira^19^, para aferição de exposição à violência comunitária, consistindo-se apenas em cinco itens que avaliam a ocorrência de ameaça ou humilhação, socos ou surra, agressão com objetos, toque íntimo forçado e estupro, o que é uma caracterização muito restrita de violência comunitária.

Assim, este estudo propôs-se a traduzir e adaptar para o português brasileiro o instrumento de aferição de exposição à violência comunitária *My Exposure to Violence (My* ETV*)*^20,21^. O *My* ETV foi desenvolvido originalmente para utilização em Chicago, EUA^20^, tendo sido posteriormente adaptado para melhor aferir especificamente a exposição à violência comunitária^21^, sendo composto por itens dicotômicos (respostas sim/não) e aborda a EVC nos âmbitos (1) vitimização direta, (2) testemunhar e (3) tomar conhecimento de episódio de violência, dividindo-se assim em três subescalas, a primeira com sete itens e as outras com oito itens cada. É considerado um dos instrumentos de aferição de exposição à violência com validação psicométrica mais robusta, um dos poucos a avaliar as três categorias de exposição^3^ e vem sendo amplamente utilizado nos EUA^11,22^ e em outros países^23,24^.

Em estudo de validação, o instrumento foi aplicado em 1.871 crianças e adolescentes de nove a 19 anos de idade, sendo encontrado um alfa de Cronbach de 0,82; uma análise fatorial confirmatória indicou a adequação da hipótese de subdivisão da exposição à violência comunitária em três vias de exposição; por fim, o escore latente de EVC, calculado por aplicação de um modelo da teoria de resposta ao item, apresentou fortes correlações com variáveis demonstradas na literatura como associadas à EVC, como medidas de ansiedade/depressão, delinquência, agressividade e características sociodemográficas^21^.

## MÉTODOS

Trata-se de um estudo psicométrico que consistiu na tradução e adaptação transcultural da versão autoaplicável do instrumento de aferição de exposição à violência comunitária, *My* ETV. A utilização do *My* ETV neste trabalho foi autorizada por um de seus autores principais.

O processo de tradução e adaptação transcultural foi baseado no modelo proposto por Beaton et al.^25^, consistindo nas seguintes etapas: (I) tradução inicial, (II) síntese das traduções, (III) retrotradução, (IV) pré-adaptação transcultural, (V) revisão por comitê de juízes, (VII) pré-teste e (VIII) submissão aos autores da versão original, tendo sido a etapa IV incluída pelos autores do presente artigo.

Duas traduções do *My* ETV (T1 e T2) para o português foram realizadas independentemente por dois tradutores com língua nativa português, bilíngues (etapa I), um deles informado quanto aos objetivos e conceitos abordados pelo instrumento e o outro era um tradutor literário profissional inglês-português, sem experiência em pesquisa na área de saúde (tradutor “ingênuo”). Finalizadas T1 e T2, os tradutores realizaram uma videoconferência, mediada por observadora externa com experiência em tradução e adaptação transcultural, para discutir as diferenças entre as traduções e chegar a uma versão síntese (T12) (etapa II). Em seguida, T12 foi enviada para dois tradutores profissionais com língua materna inglesa (um sul-africano e um americano, residentes no Brasil há sete e 26 anos, respectivamente), sem *background* da área da saúde e que desconheciam o instrumento original, para o processo de retrotradução, produzindo as versões BT1 e BT2 (etapa III).

Antes da etapa de revisão por comitê de juízes, acrescentando uma etapa ao método proposto por Beaton et al^25^. os autores realizaram uma revisão da versão síntese T12 (etapa IV), efetuando algumas modificações, tais como alterações no ordenamento das palavras, buscando aproximar o sujeito do verbo e modificações de vocabulário e expressões idiomáticas. Esse procedimento objetivou facilitar o entendimento do questionário pelo seu público-alvo e otimizar o processo de revisão pelos juízes. A nova revisão foi intitulada de pré-adaptação transcultural (pré-ATC).

Terminada a fase de traduções, ocorreu a revisão por comitê de juízes (etapa V). Foram enviados convites via e-mail para 45 potenciais juízes, os quais preenchiam pelo menos um dos seguintes critérios: (1) ter formação em Língua Inglesa e/ou Linguística; (2) ser pesquisador com experiência em desenvolvimento e/ou adaptação transcultural de instrumentos de pesquisa (metodologista), com domínio autorrelatado de língua inglesa e (3) ser pesquisador com experiência na área de violência, com domínio autorrelatado de língua inglesa.

Para cada juiz que aceitou participar do estudo foram enviados via e-mail o termo de consentimento livre e esclarecido (TCLE), as instruções para avaliação, o questionário em suas versões original, T1, T2, T12, BT1, BT2 e pré-ATC; e o instrumento de avaliação quanto às equivalências semântica, idiomática, cultural e conceitual da versão pré-ATC do *My* ETV. O instrumento de avaliação pelos juízes, em formato de documento de texto do software Microsoft Office 365^®^, consistiu na versão pré-ATC ao lado da original, com cada item com um campo de pontuação específico para os quatro tipos de equivalência, pontuados em uma escala tipo Likert com as seguintes opções: 1 = nenhuma equivalência, 2 = pouca equivalência, 3 = média equivalência, 4 = muita equivalência e 5 = total equivalência. Foi propiciado espaço para sugestões, utilizado em itens com pontuação menor que quatro. Os juízes foram instruídos a considerar equivalência semântica como a manutenção do significado das palavras entre o instrumento original e o traduzido, equivalência idiomática como a equivalência de coloquialismos ou expressões idiomáticas, equivalência cultural como a coerência da tradução com a cultura da população alvo e equivalência conceitual como a avaliação se os termos traduzidos representam o mesmo conceito dos termos originais^26^.

A concordância entre os juízes em relação à adequação das equivalências de cada item foi avaliada por meio do índice de validade de conteúdo (IVC), um dos métodos mais utilizados^27^, com a fórmula: (número de juízes que pontuaram 4 ou 5) / (nº total de juízes), a partir da qual foram calculados o IVC de cada item para cada uma das quatro equivalências e o IVC geral de cada item (IVC-I), como a média aritmética dos IVC das quatro equivalências. Por fim, foi calculado o IVC médio do instrumento (IVC-M), como a média aritmética dos IVC-I de cada um dos itens. Considerou-se um IVC-I ≥ 0,78 como excelente e um IVC-M ≥ 0,80 como aceitável e ≥ 0,90 como excelente^28^. As sugestões dos juízes foram então compiladas e avaliadas pelos autores, que conseguiram consenso sobre quais alterações realizar, chegando assim à versão pré-final, utilizada na etapa seguinte.

O pré-teste (etapa VI) foi realizado com adolescentes matriculados em uma instituição federal que oferece cursos técnicos integrados e superiores em Fortaleza, no Ceará. A escolha dessa população deu-se devido ao perfil sociocultural heterogêneo de seus estudantes, possibilitando a avaliação do instrumento por diferentes estratos da população alvo. Os participantes foram selecionados aleatoriamente entre todos os estudantes com idade até 19 anos à época do estudo (adolescentes). Os sorteados receberam por e-mail um link para um questionário, contendo TCLE (maiores de 18 anos) ou Termo de Assentimento e TCLE para o responsável (menores de 18 anos), e o instrumento de coleta de dados propriamente dito, composto pela versão pré-final do *My* ETV em português, avaliação sobre a compreensão de cada item, uma avaliação do instrumento como um todo e perguntas para caracterização sociodemográfica. Para avaliação da compreensão dos itens individualmente, cada um deles foi exposto separadamente e oferecidas aos participantes três opções de resposta: “eu não entendi a pergunta”, “eu entendi mais ou menos a pergunta” e “eu entendi a pergunta”; os participantes foram solicitados a indicar como reescreveriam os itens assinalados com a primeira ou segunda opções ou fazer sugestões, sendo fornecido campo específico para tal. O link foi enviado para 800 estudantes, objetivando-se um quantitativo de 30 a 40 participantes, no período de 8 de fevereiro de 2021 a 8 de abril de 2021.

As sugestões do público-alvo foram então compiladas e avaliadas pelos autores. A versão final foi então submetida, com toda a documentação produzida ao longo de todas as etapas, ao autor do instrumento original (etapa VII).

O estudo seguiu os princípios éticos de pesquisa envolvendo seres humanos da Resolução 466/2012 do Conselho Nacional de Saúde e foi aprovado pelos comitês de ética em pesquisa da Universidade Federal do Ceará e do Instituto Federal de Educação, Ciência e Tecnologia do Ceará sob os pareceres nº 4.026.654 (CEP/UFC) e 4.328.491 (CEP/IFCE).

## RESULTADOS

Para a elaboração da versão síntese (T12), a estruturação dos itens em quase todos os casos se deu por meio da combinação das duas traduções fornecidas. A discussão entre os tradutores iniciou-se com a padronização dos itens, ou seja, garantir que as expressões utilizadas mais de uma vez fossem traduzidas sempre da mesma forma. Nesse sentido, 21 dos 23 itens contêm a expressão “*in your whole life*” e todos contêm o termo “*ever*”. O tradutor 1 escolheu seguir literalmente o original, utilizando para o item 1, por exemplo, “Em toda sua vida, você alguma vez…”. Já o tradutor 2 compactou a expressão, utilizando “Você alguma vez na vida”. Para a síntese, considerou-se que a versão compactada mantinha o significado do original, com a vantagem de tornar a leitura menos cansativa.

Apenas uma divergência semântica relevante foi encontrada entre T1 e T2. No item 1, o trecho “*could really get hurt*” foi traduzido como “poderia se machucar seriamente” pelo tradutor 1 e como “realmente corria risco de se machucar” pelo tradutor 2. Na síntese, os tradutores concluíram que a solução presente em T2 mostrava-se mais adequada a intenção do item original, ou seja, o termo “*really*” indicando alta probabilidade de sofrer algum dano e não a gravidade do dano.

As retrotraduções apresentaram algumas diferenças em relação ao original, por exemplo, nenhum item retrotraduzido foi iniciado por “*In your whole life*”, o que já era esperado a partir das decisões tomadas na construção de T12. Ainda assim, os itens retrotraduzidos refletiram o mesmo conteúdo dos itens da versão original, tendo as retrotraduções cumprido o objetivo de checagem de validade e detecção de possíveis erros conceituais e inconsistências grosseiras em T12^25^.

Na etapa de pré-ATC, os autores realizaram alterações em 18 dos 23 itens (78,26%), tais como a substituição da expressão “Você alguma vez na vida viu” por “Alguma vez na vida você viu” e do termo “bastão” por “pedaço de pau”.

Do total de 45 profissionais convidados para a composição do comitê, 15 aceitaram participar, porém apenas 8 devolveram o instrumento de avaliação no prazo estipulado. Os juízes participantes eram profissionais das áreas de Enfermagem, Fisioterapia, Psicologia e Língua Inglesa, todos doutores e docentes, dos quais três preencheram o critério de inclusão “a”, um deles atendia ao critério “b”, dois ao critério “c”, e dois preencheram simultaneamente aos critérios “b” e “c”. Na avaliação pelo comitê de juízes, a versão pré-ATC apresentou excelentes equivalências semântica, idiomática, conceitual e cultural aceitável. Apenas um item (22) apresentou IVC-I < 0,78. O IVC-M do instrumento foi 0,92, considerado excelente (Tabela 1).

**Tabela 1:** Índices de validade de conteúdo por equivalências, por itens e total. Fortaleza, Ceará, Brasil, 2021.

	Semântica	Idiomática	Cultural	Conceitual	IVC-I
Item 1	1	1	1	1	1
Item 2	1	1	1	1	1
Item 3	1	1	0,88	1	0,97
Item 4	1	1	0,88	1	0,97
Item 5	1	1	1	0,88	0,97
Item 6	1	1	0,88	1	0,97
Item 7	0,88	1	0,88	0,88	0,91
Item 8	0,88	1	0,75	0,88	0,88
Item 9	0,88	0,88	0,75	1	0,88
Item 10	1	1	0,75	1	0,94
Item 11	0,88	0,88	0,88	0,88	0,88
Item 12	1	1	0,75	0,88	0,91
Item 13	0,88	1	0,88	1	0,94
Item 14	0,88	1	0,88	1	0,94
Item 15	1	1	1	1	1
Item 16	0,88	1	1	1	0,97
Item 17	1	1	1	1	1
Item 18	1	1	1	1	1
Item 19	0,88	0,88	0,75	0,88	0,84
Item 20	0,88	0,88	0,75	0,88	0,84
Item 21	0,75	0,88	0,75	0,75	0,78
Item 22	0,75	0,88	0,63	0,75	0,75
Item 23	0,88	0,88	0,75	0,75	0,81
Total instrumento	0,93	0,96	0,86	0,93	0,92

IVC-I: índice de validade de conteúdo para cada item.

As sugestões dos juízes deram-se majoritariamente no sentido de se obter uma maior equivalência cultural. Dos oito juízes, apenas dois não sugeriram nenhuma modificação no instrumento. O item 22, por ter obtido IVC-I < 0,78, seria o único a necessitar obrigatoriamente ser modificado, porém, devido à elevada pertinência das sugestões, 19 dos 23 itens (82,60%) sofreram alguma modificação na versão pré-final, utilizada no pré-teste. O Quadro 1 apresenta as versões originais, pré-ATC e pré-final do *My* ETV.

**Quadro 1:** Versões original, pré-ATC e final do *My Exposure to Violence* . Fortaleza, Ceará, Brasil, 2021.

Original	Pré-ATC	Versão final
1. *In your whole life, have you EVER seen someone else get chased when you thought they could really get hurt?*	Alguma vez na vida você viu alguma outra pessoa ser perseguida e achou que ela realmente corria risco de se machucar? (=)	Alguma vez na vida você viu alguma outra pessoa ser perseguida e achou que ela realmente corria risco de se machucar? (=)
2. *In your whole life, have you EVER been chased when you thought that you could really get hurt?*	Alguma vez na vida você foi perseguido e achou que realmente corria risco de se machucar?	Alguma vez na vida você foi **perseguido(a)** e achou que realmente corria risco de se machucar?
3. *In your whole life, have you EVER seen someone else get hit, slapped, punched, or beaten up? This does not include when they were playing or fooling around.*	Alguma vez na vida você viu alguma outra pessoa apanhar, levar tapa, soco ou surra? Isso não inclui situações de brincadeira ou gozação.	Alguma vez na vida você viu alguma outra pessoa apanhar, levar um tapa, **um soco ou uma surra?** *Não considerar situações em que os envolvidos estão rindo ou brincando.*
4. *In your whole life, have you EVER been hit, slapped, punched, or beaten up? Again, this does not include when you were playing or fooling around.*	Alguma vez na vida você apanhou, levou tapa, soco ou surra? Mais uma vez, isso não inclui situações de brincadeira ou gozação.	Alguma vez na vida você apanhou, **levou um tapa, um soco ou uma surra?** Mais uma vez*, não considerar situações em que os envolvidos estão rindo ou brincando.*
5. *In your whole life, have you EVER seen someone else get attacked with a weapon, like a knife or bat? This does not include getting shot or shot at.*	Alguma vez na vida você viu alguma outra pessoa ser atacada com uma arma, como uma faca ou pedaço de pau? Isso não inclui ser baleado ou ter tiros disparados contra si.	Alguma vez na vida você viu alguma outra pessoa ser atacada com uma arma, como uma faca ou pedaço de pau? **Isso não inclui a pessoa ser baleada ou ter tiros disparados contra ela.**
6. *In your whole life, have you EVER been attacked with a weapon, like a knife or bat? Again, this does not include getting shot or shot at.*	Alguma vez na vida você foi atacado com uma arma, como uma faca ou um pedaço de pau? Mais uma vez, isso não inclui ser baleado ou ter tiros disparados contra si.	Alguma vez na vida você foi **atacado(a)** com uma arma, como uma faca ou um pedaço de pau? Mais uma vez, isso não inclui ser baleado ou ter tiros disparados contra si.
7. *In your whole life, have you EVER seen someone else get shot? This doesn’t include seeing someone shot with a BB gun or any type of toy gun.*	Alguma vez na vida você viu alguma outra pessoa levar um tiro? Isso não inclui ver alguém levar um tiro de arma de chumbinho ou de qualquer tipo de arma de brinquedo. (=)	Alguma vez na vida você viu alguma outra pessoa levar um tiro? Isso não inclui ver alguém levar um tiro de arma de chumbinho ou de qualquer tipo de arma de brinquedo. (=)
8. *In your whole life, have you EVER been shot? Again, this doesn’t include being shot with a BB gun or any type of toy gun.*	Alguma vez na vida você levou um tiro? Mais uma vez, isso não inclui levar um tiro de arma de chumbinho ou de qualquer tipo de arma de brinquedo. (=)	Alguma vez na vida você levou um tiro? Mais uma vez, isso não inclui levar um tiro de arma de chumbinho ou de qualquer tipo de arma de brinquedo. (=)
9. *In your whole life, have you EVER seen someone else get shot AT, but not actually wounded?*	Alguma vez na vida você viu atirarem em alguma outra pessoa, mas sem essa pessoa ser mesmo ferida?	Alguma vez na vida você viu atirarem em alguma outra pessoa, mas sem essa pessoa **ter sido ferida?**
10. *In your whole life, have you EVER been shot AT, but not actually wounded?*	Alguma vez na vida atiraram em você, mas sem você ter sido mesmo ferido?	Alguma vez na vida atiraram em você, mas **sem você ter sido ferido(a)?**
11. *In your whole life, have you EVER seen someone else get killed as a result of violence, like being shot, stabbed, or beaten to death?*	Alguma vez na vida você viu alguma pessoa ser morta por meio de violência, como levar um tiro, ser esfaqueada ou espancada até a morte?	Alguma vez na vida você viu alguma pessoa ser morta **como consequência** de violência, como levar um tiro, ser esfaqueada ou espancada até a morte?
12. *In your whole life, have you EVER been sexually assaulted, molested, or raped?*	Alguma vez na vida você sofreu abuso sexual, foi molestado ou estuprado?	Alguma vez na vida você sofreu abuso sexual, foi **molestado(a) ou estuprado(a)** *ou lhe tocaram nas partes íntimas sem que você quisesse?*
13. *OTHER THAN WHAT YOU HAVE ALREADY TOLD ME, have your EVER seen someone threaten to seriously hurt another person? This includes being threatened with a weapon.*	ALÉM DO QUE VOCÊ JÁ me CONTOU, alguma vez na vida você viu alguém ameaçar machucar seriamente outra pessoa? Isso inclui ser ameaçado com uma arma.	ALÉM DO QUE VOCÊ JÁ me CONTOU, alguma vez na vida você viu alguém ameaçar ou machucar seriamente outra pessoa? Isso inclui *ameaça com qualquer tipo de arma.*
14. *OTHER THAN WHAT YOU HAVE ALREADY TOLD ME, has someone EVER threatened to seriously hurt you? Again, this includes being threatened with a weapon.*	ALÉM DO QUE VOCÊ JÁ me CONTOU, alguma vez na vida alguém ameaçou machucar você seriamente? Mais uma vez, isso inclui ser ameaçado com uma arma.	ALÉM DO QUE VOCÊ JÁ me CONTOU, alguma vez na vida alguém ameaçou machucar você seriamente? Mais uma vez, isso inclui *ameaça com qualquer tipo de arma.*
15. *In your whole life, have you EVER been told that someone you knew had been shot, but not killed?*	Alguma vez na vida lhe contaram que alguém que você conhecia tinha levado um tiro, mas não foi morto?	Alguma vez na vida **te** contaram que alguém que você conhecia tinha levado um tiro, mas **não tinha morrido?**
16. *In your whole life, have you EVER been told that someone you knew had been killed?*	Alguma vez na vida lhe contaram que alguém que você conhecia tinha sido assassinado?	Alguma vez na vida **te** contaram que alguém que você conhecia tinha sido **assassinado(a)?**
17. *In your whole life, have you EVER been told that someone you knew had been raped?*	Alguma vez na vida lhe contaram que alguém que você conhecia tinha sido estuprado(a)? (=)	Alguma vez na vida te contaram que alguém que você conhecia tinha sido estuprado(a)? (=)
18. *In your whole life, have you EVER seen someone else sexually assaulted, molested, or raped?*	Alguma vez na vida você viu alguma outra pessoa sofrer abuso sexual, ser molestado ou estuprado?	Alguma vez na vida você viu alguma outra pessoa sofrer abuso sexual, ser **molestado(a)** ou **estuprado(a)** *ou tocarem nas partes íntimas dela sem que ela quisesse*?
19. *In your whole life, have you EVER been told that someone you knew had been chased when you thought they could really get hurt.*	Alguma vez na vida lhe contaram que alguém que você conhecia tinha sido perseguido e achou que essa pessoa realmente correu risco de se machucar?	Alguma vez na vida **te** contaram que alguém que você conhecia tinha sido **perseguido(a) e você achou que essa pessoa realmente poderia se machucar?**
20. *In your whole life, have you EVER been told that someone you knew had been slapped, punched, or beaten up? This does not include when they were playing or fooling around.*	Alguma vez na vida lhe contaram que alguém que você conhecia tinha levado tapa, soco ou surra? Isso não inclui situações de brincadeira ou gozação.	Alguma vez na vida **te** contaram que alguém que você conhecia tinha levado **um tapa, um soco ou uma surra?** *Não considerar situações em que os envolvidos estão rindo ou brincando.*
21. *In your whole life, have you EVER been told that someone you knew had been attacked with a weapon, like a knife or bat? This does not include getting shot or shot at.*	Alguma vez na vida lhe contaram que alguém que você conhecia tinha sido atacado com uma arma, como uma faca ou pedaço de pau? Isso não inclui ser baleado ou ter tiros disparados contra si.	Alguma vez na vida **te** contaram que alguém que você conhecia tinha sido **atacado(a)** com uma arma, como uma faca ou pedaço de pau? Isso não inclui ser **baleado(a)** ou **ter tiros disparados contra ela.**
22. *In your whole life, have you EVER been told that someone you knew had been shot AT, but not actually wounded?*	Alguma vez na vida lhe contaram que atiraram em alguém que você conhecia, mas sem essa pessoa ser mesmo ferida?	Alguma vez na vida te contaram que atiraram em alguém que você conhecia, **mas sem essa pessoa ter sido ferida?**
23. *OTHER THAN WHAT YOU HAVE ALREADY TOLD ME, in your whole life, have you EVER been told that someone had threatened to seriously hurt someone you knew? This includes being threatened with a weapon.*	ALÉM DO QUE VOCÊ JÁ me CONTOU, alguma vez na vida lhe contaram que alguém havia ameaçado machucar seriamente alguém que você conhecia? Isso inclui ameaças com arma.	ALÉM DO QUE VOCÊ JÁ me CONTOU, alguma vez na vida **te** contaram que **alguma pessoa** havia ameaçado machucar seriamente alguém que você conhecia? Isso inclui *ameaça com qualquer tipo de arma.*

Pré-ATC: pré-adaptação transcultural.

Nota: na versão final, negrito indica sugestão acatada literalmente e *itálico* indica alteração realizada pelos autores de modo a incorporar sugestão. O sinal (=) indica que não foram realizadas alterações.

A formatação em caixa alta foi mantida apenas para a expressão “ALÉM DO QUE VOCÊ JÁ me CONTOU” (itens 13, 14 e 23) por opção dos autores.

A média de idade dos 39 participantes do pré-teste foi de 17,48 anos (DP = 1,27), com mínimo de 15 e máximo de 19 anos. A maioria era do sexo feminino (51,28%), parda (71,79%), católica (41,03%), solteira (92,31%) e residia em Fortaleza (74,36%).

Dos 23 itens da versão pré-final, 21 (91,30%) foram totalmente compreendidos por mais de 90% dos participantes. Os itens com maior dificuldade de compreensão foram os itens nove e 10, contudo ambos foram relatados como totalmente compreendidos por 35 estudantes (89,74%), conforme demonstra a Tabela 2.

**Tabela 2:** Compreensão dos itens do Minha Exposição à Violência. Fortaleza, Ceará, Brasil, 2021.

Item	Eu não entendi a pergunta n (%)	Entendi mais ou menos a pergunta n (%)	Eu entendi a pergunta n (%)
1	0	0	39 (100)
2	0	1 (2,56)	38 (97,44)
3	0	2 (5,13)	37 (94,87)
4	0	0	39 (100)
5	0	0	39 (100)
6	0	1 (2,56)	38 (97,44)
7	0	0	39 (100)
8	0	0	39 (100)
9	2 (5,13)	2 (5,13)	35 (89,74)
10	2 (5,13)	2 (5,13)	35 (89,74)
11	0	0	39 (100)
12	0	0	39 (100)
13	0	1 (2,56)	38 (97,44)
14	0	1 (2,56)	38 (97,44)
15	0	0	39 (100)
16	1 (2,56)	0	38 (97,44)
17	0	0	39 (100)
18	0	0	39 (100)
19	0	2 (5,13)	37 (94,87)
20	0	0	39 (100)
21	0	1 (2,56)	38 (97,44)
22	0	0	39 (100)
23	0	1 (2,56)	38 (97,44)

Apenas uma pequena parcela dos participantes 6 (15,38%) fez sugestões de modificações nos itens. Dos 23 itens, oito (34,78%) receberam sugestões/críticas. O Quadro 2 apresenta todos os comentários dos participantes.

**Quadro 2:** Sugestões realizadas pelos participantes do pré-teste. Fortaleza, Ceará, 2021.

Item	Sugestões
2. Alguma vez na vida você foi perseguido(a) e achou que realmente corria risco de se machucar?	Alguma vez na vida você foi exposto(a) a algum tipo de violência, seja física ou psicológica. Ou já se sentiu agredido física ou psicologicamente?
5. Alguma vez na vida você viu alguma outra pessoa ser atacada com uma arma, como uma faca ou pedaço de pau? Isso não inclui a pessoa ser baleada ou ter tiros disparados contra ela.	Eu entendi, porém colocaria a observação em parênteses.
9. Alguma vez na vida você viu atirarem em alguma outra pessoa, mas sem essa pessoa ter sido ferida?	Alguma vez na vida você viu tentarem atirar numa pessoa, mas, por não terem a acertado, a pessoa não foi ferida? Você já presenciou uma tentativa de homicídio por arma de fogo?
10. Alguma vez na vida atiraram em você, mas sem você ter sido ferido(a)?	Alguma vez na vida tentaram atirar em você, mas, por não terem acertado, você não foi ferido? Você já passou por uma experiência traumática, onde tentaram te ferir com uma arma de fogo?
13. ALÉM DO QUE VOCÊ JÁ me CONTOU, alguma vez na vida você viu alguém ameaçar ou machucar seriamente outra pessoa? Isso inclui ameaça com qualquer tipo de arma.	Entendi o que quis dizer, porém não contei nada, logo não poderia ser “ALÉM DO QUE VOCÊ JÁ ME CONTOU”.
14. ALÉM DO QUE VOCÊ JÁ me CONTOU, alguma vez na vida alguém ameaçou machucar você seriamente? Mais uma vez, isso inclui ameaça com qualquer tipo de arma.	Entendi o que quis dizer, porém não contei nada, logo não poderia ser “ALÉM DO QUE VOCÊ JÁ ME CONTOU”.
19. Alguma vez na vida te contaram que alguém que você conhecia tinha sido perseguido(a) e você achou que essa pessoa realmente poderia se machucar?	Alguma vez na vida te contaram que alguém que você conhecia tinha sido perseguido(a) e você achou que essa pessoa poderia ter se machucado?
23. ALÉM DO QUE VOCÊ JÁ me CONTOU, alguma vez na vida te contaram que alguma pessoa havia ameaçado machucar seriamente alguém que você conhecia? Isso inclui ameaça com qualquer tipo de arma.	Entendi o que quis dizer, porém não contei nada, logo não poderia ser “ALÉM DO QUE VOCÊ JÁ ME CONTOU”.

Nota: a formatação em caixa alta foi mantida apenas para a expressão “ALÉM DO QUE VOCÊ JÁ me CONTOU” (itens 13, 14 e 23) por opção dos autores.

Os autores avaliaram que as sugestões ou eram impertinentes (itens 13, 14 e 23) ou implicariam em mudança substancial no conteúdo dos itens (item 2 e item 10b) ou apresentavam uma formulação excessivamente técnica (item 9b). Em relação às demais, apesar de pertinentes, julgou-se que elas diferiam muito pouco dos itens apresentados. Sendo assim, não foram realizadas alterações nos itens da versão pré-final.

Na avaliação geral, a grande maioria dos participantes, 31 (79,49%), considerou que responderia o instrumento com facilidade, apenas 12 (30,77%) consideraram o questionário longo e apenas 11 (28,21%) o consideraram repetitivo. A maioria da amostra, 32 (82,05%) e 37 (94,87%), julgou que a partir das respostas do instrumento é possível saber se um(a) adolescente já sofreu algum tipo de violência e se um(a) adolescente conhece alguém que já sofreu algum tipo de violência, respectivamente.

Considerando a avaliação positiva do instrumento como um todo e a elevada compreensão de seus itens pelos participantes do pré-teste, considerou-se finalizado o processo de tradução e adaptação ao contexto cultural brasileiro do *My* ETV, com o título Minha Exposição à Violência, sendo mantida a versão pré-final como versão final. Todas as versões produzidas ao longo do processo foram submetidas a um dos autores originais, o qual aprovou a versão final em português e nos parabenizou por manter a intenção conceitual dos itens.

## DISCUSSÃO

A tradução e adaptação transcultural para o português brasileiro do instrumento de aferição de exposição à violência comunitária, *My* ETV, foi realizada obtendo excelentes equivalências semântica, idiomática, conceitual e aceitável equivalência cultural por juízes, além da compreensão elevada dos itens pela população alvo.

O processo metodológico de preparação de um instrumento de pesquisa para a aplicação em uma população com idioma, cultura, conceitos e experiências distintos daquela para a qual foi originariamente desenvolvido requer que seja seguido um roteiro rigoroso e padronizado para se obter um instrumento traduzido confiável^29^. Neste trabalho, foram seguidas as etapas propostas por Beaton et al.^25^, referencial utilizado no Brasil^30^ e internacionalmente^31^ na maioria dos estudos de adaptação transcultural, acrescido de uma etapa denominada pré-ATC que consistia em uma análise preliminar de aspectos linguísticos culturais.

Na etapa I, as traduções foram realizadas independentemente por dois tradutores com língua materna do idioma alvo (português brasileiro) e sintetizadas na versão T12 (etapa II). A escolha de tradutores qualificados é crucial para a obtenção de traduções de alta qualidade. A composição de tradutor informado e tradutor ingênuo é necessária para garantir que as versões traduzidas abranjam tanto aspectos técnicos quanto reflitam a língua falada e suas nuances culturais^29^.

A retrotradução funciona como uma verificação de controle de qualidade adicional, tendo o objetivo de verificar se a versão síntese reflete o conteúdo do instrumento original, não pressupondo que eles se mantenham literalmente idênticos, mas que mantenham a equivalência conceitual^32^. Na etapa III, as retrotraduções não apresentaram grandes discrepâncias entre si e nem em relação ao original.

Ocorreu a adição de uma etapa ao roteiro proposto por Beaton et al.^25^, intitulada pré-ATC, consistindo na revisão da versão síntese T12 (etapa IV) antes da revisão pelo comitê de juízes. Acréscimos e modificações em processos de adaptação transcultural são comuns e, em geral, bem aceitos, desde que sejam reconhecidos e descritos em detalhes^33^.

Na etapa V, o comitê de juízes atingiu a composição mínima sugerida por Beaton et al.^25^, incluindo linguistas, metodologistas e especialistas na área do construto, sendo formado por profissionais de elevada expertise, o que possibilitou que fossem geradas sugestões de elevada pertinência, as quais foram acatadas sempre que possível, visando obter a maior relevância e compreensibilidade possível dos itens, garantindo-se assim um instrumento bem adaptado ao contexto brasileiro.

Esse processo refletiu-se positivamente na etapa de pré-teste. Poucas sugestões foram feitas e, considerando-se a alta taxa de compreensão dos itens, não foram realizadas novas alterações no instrumento. A aplicação a uma amostra com diferentes perfis de adolescentes deu garantia aos resultados de avaliação da compreensão do instrumento. Além disso, a maioria dos participantes não considerou o instrumento nem longo nem repetitivo, o que é relevante pois esses são fatores que podem estar associados a uma menor taxa de resposta^34^. Assim, o *My* ETV foi traduzido e adaptado transculturalmente para o português brasileiro, o que é o primeiro passo para uma futura validação do instrumento e aplicação em amostras maiores de adolescentes.

As contribuições deste estudo são relevantes tendo em vista a escassez de instrumentos devidamente adaptados para o português na área de violência comunitária. Alguns trabalhos avaliaram EVC por meio de outros instrumentos, porém geralmente não há descrições dos processos de tradução e adaptação transcultural e/ou os instrumentos utilizados não abrangem o conceito de violência comunitária em suas três vias de exposição (vitimização direta, testemunhar e tomar conhecimento de episódio de violência).

Não foi localizada nenhuma pesquisa que aplicou qualquer instrumento de aferição de exposição à violência comunitária em adolescentes no nordeste do Brasil. Salienta-se ainda que o *My* ETV é um dos poucos instrumentos a avaliar as três categorias de EVC, e que, até a data de finalização deste trabalho não foi localizado estudo de adaptação transcultural do *My* ETV realizado no Brasil ou nenhuma versão portuguesa do instrumento, ressaltando sua contribuição em disponibilizar a versão brasileira do *My* ETV para uso e validação nos mais variados contextos de pesquisas.

Como limitações deste estudo, pode-se citar a coleta dos dados de forma virtual. Tanto na etapa de avaliação por juízes como no pré-teste encontros presenciais poderiam ter gerado uma maior riqueza de informações, possibilidade inviabilizada pelo contexto da pandemia de SARS-CoV-2, porém acreditamos que os benefícios da coleta virtual, como baixo custo, otimização de tempo e oportunidade para responder o questionário em momento escolhido, com maior tempo para reflexão, superaram as possíveis limitações.

## CONCLUSÕES

A violência comunitária é um problema de saúde pública que impacta principalmente os adolescentes, porém não havia um instrumento adequado em língua portuguesa para aferição da exposição à violência comunitária. O presente estudo realizou a tradução e adaptação transcultural para o português brasileiro do instrumento de aferição *My* ETV, seguindo o protocolo mais recomendado e utilizado na literatura mundial. Outras propriedades de medidas, como confiabilidade e validades de critério e de construto, devem ser avaliadas em estudos posteriores para fortalecer as evidências da tradução e adaptação para o português brasileiro.
